# UNFAIR TREATMENT, SOCIAL COHESION, AND COGNITION IN BLACK ADULTS LIVING IN URBAN NEIGHBORHOODS

**DOI:** 10.1093/geroni/igad104.1189

**Published:** 2023-12-21

**Authors:** Erica Fan, Tamara Dubowitz, Wendy Troxel, Andrea Weinstein, Tiffany Gary-Webb, Bonnie Ghosh-Dastidar, Jennifer Manly, Andrea Rosso

**Affiliations:** University of Pittsburgh, School of Public Health, Pittsburgh, Pennsylvania, United States; RAND Corporation, Pittsburgh, Pennsylvania, United States; RAND Corporation, Pittsburgh, Pennsylvania, United States; University of Pittsburgh, Pittsburgh, Pennsylvania, United States; University of Pittsburgh, Graduate School of Public Health, Pittsburgh, Pennsylvania, United States; RAND Corporation, Pittsburgh, Pennsylvania, United States; Columbia University Irving Medical Center, New York City, New York, United States; University of Pittsburgh, Pittsburgh, Pennsylvania, United States

## Abstract

Exposure to discrimination has been associated with poorer cognitive function but this association may be influenced by broader social factors, such as neighborhood social cohesion. We investigated the associations between unfair treatment, social cohesion, and cognitive function in 234 participants ≥50 years, recruited from urban, primarily Black neighborhoods in Pittsburgh, PA. Neuropsychological assessments were conducted in 2018 to create six cognitive domain z-scores, adjusted for age, sex, and education level. Social cohesion was assessed in 2013 and 2018. Change in social cohesion scores over time categorized participants into four groups: consistently high, consistently low, increasing, and decreasing scores. Unfair treatment was assessed via questionnaire in 2018. Adjusted generalized linear models assessed associations between unfair treatment and cognition. Unfair treatment was significantly associated with executive function (β=-0.041, p=0.01) and marginally associated with language (β=-0.030, p=0.07). Associations persisted with change in social cohesion in the models and we found that social cohesion moderated the association between unfair treatment and executive function (p=.06). In those with consistently low social cohesion, more unfair treatment was associated with worse executive function (β=-0.085, p=0.02). Both increasing and decreasing social cohesion showed a similar negative association between unfair treatment and executive function at a trend level (β=-0.122, p=0.08 and β=-0.082, p=0.10 respectively). There was no association for those with consistently high social cohesion. Individuals experiencing unfair treatment and consistently low levels of neighborhood social cohesion had lower executive function. These findings highlight the importance of intervening at multiple levels and addressing both interpersonal discrimination and structural sources.

